# Radiographic and Clinical Findings of Single-Visit Root Canal Treatments with Apical Enlargement in Necrotic Teeth: A Retrospective Cohort Study

**DOI:** 10.1155/2020/7912638

**Published:** 2020-09-28

**Authors:** Tan F. Eyüboğlu, Keziban Olcay, Erhan Erkan, Mutlu Özcan

**Affiliations:** ^1^Faculty of Dentistry, Department of Endodontics, Istanbul Medipol University, Istanbul, Turkey; ^2^Faculty of Dentistry, Department of Endodontics, Istanbul University Cerrahpaşa, Istanbul, Turkey; ^3^Division of Dental Biomaterials, Clinic for Reconstructive Dentistry, University of Zurich, Zurich, Switzerland

## Abstract

This study evaluated the long-term clinical outcomes of single-visit root canal treatments with apical enlargement on patients with necrotic pulp tissue retrospectively. A total of 137 teeth with necrotic pulp tissue which underwent single-visit root canal treatments were included. The root canals were shaped up until the apical constriction, which was determined by an apex locator. The outcomes were evaluated by two independent and calibrated endodontists clinically and radiographically. Teeth were dichotomized into healed (PAI ≤ 2, no signs or symptoms) and nonhealed (PAI > 2, with/without signs or symptoms) groups. Each patients' preoperative PAI and lesion size were recorded to evaluate the preoperative periapical status as well as several other prognostic factors. Statistical analyses were performed (*p* = 0.05) on ninety teeth. The mean observation time was 60 months. Out of ninety teeth, 87 (96.7%) were healed and 3 (3.3%) were nonhealed. No correlations were found between the prognostic factors and the outcomes (*p* > 0.05). Cohen's kappa and Gwet's agreement coefficient scores between the preoperative PAI scores and preoperative lesion sizes showed good agreements, with values of 0.834 and 0.898, respectively. Apical enlargement is a viable treatment option for single-visit root canal treatments.

## 1. Introduction

The primary goal of endodontic therapy is to promote healing and prevent apical periodontitis in the periapical area [1] where chemomechanical cleaning is essential for this purpose [2]. In a recent study, achieving a successful root canal treatment was associated with high-quality root canal filling and postendodontic restoration and the latter was reported to be mandatory for a healthy periapical tissue [3]. The high prevalence of bacterial biofilms and the presence of anatomical complexities, such as ramifications and lateral canals, have been proven to make the apical third of the root canal a challenging area for cleaning and disinfection to achieve high-quality root canal filling. These complexities can cause both persistent infections and compromised outcomes [4]. The prevention of these poor outcomes has contributed to the creation of a sound and scientifically supported approach to apical enlargement, which can be described as the enlargement of apical third of the root canal, during root canal treatment in order to reduce the intracanal bacteria level [2] and achieve better healing results [5]. However, there is still no consensus on where the apical enlargement should end, with measurements varying from 0.5 to 1 mm short of the apex, at the apex, and beyond the apical foramen [2–6]. Moreover, the extension of apical enlargement in means of final file size has not reached a consensus either. Traditionally, using three sizes larger than first apical binding file was recommended for apical shaping [7]. However, this approach was indicated to be inadequate [8] due to the anatomy of apical region [9]. The files that bound at the working length were also reported to reflect the apical root canal diameter inaccurately [8]. To remove uniform and sufficient dentin from the root canal walls, a range of six to eight sizes larger than first apical binding file were recommended to be used [9]. However, current reports have described the deleterious effects of apical enlargement [10, 11]. One of the suggestions was that the root canal system should be shaped without widening the apical constriction in necrotic pulp cases [12]. A minimum apical preparation size around size 20 [13] with a continuous taper [14] was suggested due to damaging effect of apical enlargement. However, in an animal study, apical enlargement was reported to exhibit better results in teeth with periapical lesions [15]. Moreover, the apical enlargement of teeth decreased intracanal bacteria in humans [2]. These conflicting results constitute divergent opinions on apical enlargement in root canal treatment.

Although there is no consensus with regard to single- or multiple-visit endodontic treatments [16–19], reduced costs and treatment times, avoiding microleakage, preventing interappointment recontamination, and patient preferences [20–22] suggest that single-visit root canal treatment is a viable treatment option. In a recent randomized controlled trial, the enlargement of the root canal up to three sizes compared to four, five, and six sizes larger than first apical binding file was reported to be adequate in root canal treatments in which calcium hydroxide [Ca(OH)_2_] powder mixed with 2% chlorhexidine liquid was used as an intracanal medicament [23]. However, due to the absence of interappointment dressing in a single-visit treatment, disinfection of the apical third of the root might be a greater hurdle than that in a multiple-visit treatment. Apical enlargement ensures the removal of infected dentinal tissue as well as efficient delivery of irrigation solution to the apical third of the infected root canal by means of increased apical patency and thus achieving high clinical success rate [24]. Therefore, the aim of this retrospective cohort study was to evaluate the clinical outcomes, namely, periapical and clinical complication frequencies, and success rates, of endodontically treated necrotic teeth undergoing apical enlargements during single-visit treatments.

## 2. Materials and Methods

This study was approved by the ethics committee of the university (file number: 10840098-604.01.01-E.20703, decision number: 364). The Helsinki Declaration guidelines were followed throughout the study. All participants signed written informed consent forms prior to their admission.

### 2.1. Inclusion and Exclusion Criteria

This cohort study included all the patients who presented to the university clinic between December 2011 and January 2014 and were appointed for a single-visit orthograde root canal treatment with apical enlargement. Patients (i) with medical history contradictory to endodontic therapy and (ii) who are younger than 18 years old (teeth (a) with periodontal pocketdepth ≥ 4 mm, (b) vertical root fractures, (c) apical resorption and (d) teeth that requiring or received surgical endodontic treatments, and (e) teeth with root canals that require an initial apical file (the first file that binds at working length) larger than #15 K-file) were excluded from the study. A total of 137 necrotic teeth of 137 patients were included in this research. The indications were made according to clinical and radiological evaluation. Negative response to the cold test and the presence of sensitivity to percussion and palpation were taken into consideration for indication. Since a simple random sampling method was used to determine a power of 80% as described by Walters and the sample size was determined to be 88 with a *β* = 0.20 and *α* = 0.05, the sample size of 137 teeth was deemed satisfactory [25]. All treatments were carried out by one endodontist (T.F.E.) with 15 years of experience. Clinical examination, cold pulp testing, and periapical radiographs (Kodak RVG 5100; Carestream Health, Inc., Rochester, NY, USA) with the paralleling technique (RINN XCP-ORA, Dentsply Sirona, Bensheim, Germany) were used to establish diagnosis. The exposure dose was 1.22 mGy, and the exposure time was 0.16 seconds.

### 2.2. Treatment Procedure

Whole treatment procedure in each patient, including the root canal treatment and direct coronal restoration (with or without a post and core placement), was carried out in a single appointment under 3.5x magnification. Total treatment time for each tooth was no longer than 70 minutes. After administering 40 mg/ml of articaine hydrochloride plus 0.006 mg/ml of epinephrine hydrochloride (Ultracaine DS Forte; Aventis Pharma, Istanbul, Turkey), a rubber dam (Hygenic Dental Dam Kit; Coltene/Whaledent, Langenau, Germany) was placed to isolate the relevant tooth. All of the procedures, including carious removal, access cavity preparation, and the remainder of the root canal treatment, were carried out using rubber dam isolation. After scouting the root canal with a #10 K-file (Dentsply Maillefer, Ballaigues, Switzerland) to ensure patency, the coronal flaring was carried out using a SC1coronal flaring file (Revo-S, Micro-Méga, Besançon, France) 3 mm from the root canal orifice. The patency was maintained in all root canals prior to and during the chemomechanical cleaning. Working length was determined with an apex locator (Apex Pointer; Micro-Méga, Besançon, France) at the apical constriction. A glide path was ensured with the initial apical file (#15 K-file, Dentsply Maillefer) prior to shaping with the Revo-S files up to the apical constriction, according to the manufacturer's instructions. For irrigation of the root canals after each file, 2 ml of 2.5% NaOCl (Wizard; Rehber Chemistry, Istanbul, Turkey) was used. The apical enlargements of mesial root canals of two mandibular teeth were finished with AS35 (Revo-S, Micro-Méga, Besançon, France) while distal root canals of these two mandibular teeth as well as all the other root canals in the other 135 teeth were completed with AS40 (Revo-S, Micro-Méga). The choice of finishing with a smaller size file in these two mandibular molar teeth was made according to the anatomy of the root canal by the clinician. The Revo-S system was chosen due the unique physical properties of the AS35 and AS40 files. These files are #35 and #40, respectively, with .06 taper at the apical 5 mm of the files. The rest of both files are taperless. After the root canal shaping was finished, the final irrigation protocol was performed using 2.5 ml of 5% ethylenediaminetetraacetic acid (Wizard; Rehber Chemistry, Istanbul, Turkey) for one minute, 2.5 ml of 2.5% NaOCl for 30 seconds, and 5 ml of distilled water for 30 seconds. Gutta-percha matched to the last shaping file was used as a master cone to fill the root canal using a single-cone technique. Resin-based root canal paste (AH Plus; Dentsply DeTrey, Konstanz, Germany) was introduced to the gutta-percha master cone with a brushing motion. If necessary, accessory gutta-percha cones (Revo-S SU 25; Micro-Méga) were also used. The accessory cones were introduced into the root canal using a rotary condenser (Revo-S Condenser, Micro-Méga) according to the manufacturer's instructions.

After the completion of root canal obturation, Single Bond 2 (3M ESPE, St. Paul, MN, USA) was applied to the access cavities, based on the manufacturer's instructions, using a total-etch technique prior to applying the flowable resin composite (Filtek Ultimate; 3M ESPE) to the root canal orifices. The coronal restorations were then completed using a universal resin composite restorative material (Filtek Supreme Ultra, 3M ESPE) with incremental technique. If a post core was needed, a fiber post (Cytec Blanco, HT-Glasfiber; E. Hahnenkratt GmbH, Königsbach-Stein, Germany) and composite core (RelyX U200, 3M ESPE) were placed according to the manufacturer's instructions prior to the fixed prosthetic restoration, depending on the prosthetic plan. All prosthetic restorations were completed within two weeks of the root canal treatment.

### 2.3. Follow-Up Evaluation

**Figure 1:**
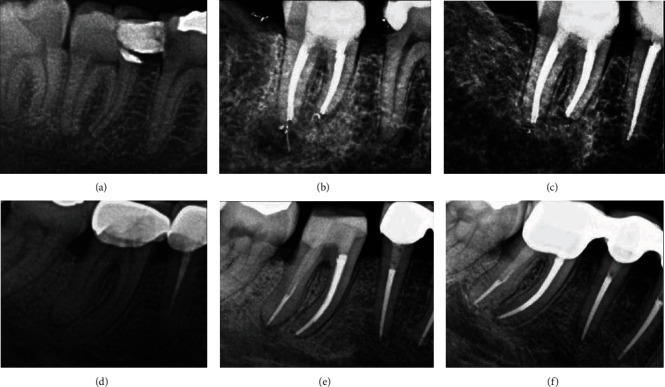
Radiographic evaluation of different teeth before and after the treatment: (a) right mandibular second molar tooth, preoperative radiograph; (b) right mandibular second molar tooth, after the treatment; (c) right mandibular second molar tooth, a 68-month follow-up; (d) right mandibular first molar tooth, preoperative radiograph; (e) right mandibular first molar tooth, after the treatment; (f) right mandibular first molar tooth, a 50-month follow-up.

Using a paralleling technique, periapical radiographs of the relevant tooth were made immediately after the treatment and during the follow-up Figure 1.

Clinical evaluation was made to record any clinical sign and symptoms such as pain, sensitivity to percussion and palpation, swelling, and the presence of fracture. The preoperative (gender, age at treatment, preoperative pain, tooth type, radiolucency, the preoperative PAI scores, and periodontal defects), intraoperative (root filling length, root filling voids, complications, and sealer extrusion), and postoperative (density of root fill, quality of coronal restoration, postoperative sign and symptoms, radiolucency, the postoperative PAI scores, fracture, restoration at follow-up, and the presence of post) data were recorded as well as the preoperative and postoperative radiographs (Table 1). The lesion size measurements were completed on the periapical radiographs using Kodak RVG 5100 digital program (Carestream Health, Inc., Rochester, NY, USA).

All the data, including the clinical signs, symptoms, and follow-up radiographs, were recorded and evaluated by the coexaminers (K.O. and E.E.). For each tooth treated, the periapical index (PAI) scores, as described by Ørstavik et al. [26], were recorded at the baseline and during the follow-up.

### 2.4. Observer Calibration

A paralleling technique was used for all the radiographs that were assigned for the PAI score evaluation. Prior to the evaluation, one hundred reference radiographs were used to calibrate the investigators, as described previously [16]. The calibration process was carried out twice with a two-month interval, and both the intraexaminer and interexaminer kappa values were recorded. The kappa scores ranged between 0.936 and 0.987 (Table 2). The PAI scores were dichotomized into healed (PAI ≤ 2, no sign or symptoms) and nonhealed (PAI > 2, with or without sign or symptoms) groups as described previously [19, 20]. The multiple-rooted teeth were scored according to the root apex with the highest PAI score [19, 20]. If the evaluators reported different scores, the worst score was recorded [27].

### 2.5. Statistical Analyses

The Number Cruncher Statistical System (2007; NCSS Statistical Software, Kaysville, Utah, USA) was used for the statistical analyses. The descriptive statistics, such as the mean, standard deviation, median, first quartile, third quartile, minimum, maximum, frequency, and percentage values, were reported in tables. An independent sample *t*-test was used to compare the normally distributed variables between the groups. The Kruskal-Wallis test was used to compare the variables between the groups, and the Dunn-Bonferroni test was used as a post hoc test. The associations among the nominal variables were tested via the Fisher exact test and Fisher-Freeman-Halton exact test. The statistical significance was determined at the *p* < 0.05 level.

## 3. Results

The treatments of 137 necrotic teeth were completed between December 2011 and January 2014 in 137 patients. The apical enlargements of mesial root canals of two mandibular teeth were finished with AS35 while the others were completed with AS40. Ninety of these (65.6%) agreed to further follow-ups, while 47 patients (34.4%) were lost to follow-up, mostly because they did not respond (32 patients, 23.3%) or declined to come to the clinic due to time constraints in their daily schedules combined with no problems or symptoms with regard to their respective teeth (15 patients, 11.1%) (Table 1). Among the prognostic factors, there were no statistically significant differences between the inception cohort and the study group (*p* > 0.05) (Table 1). The observation time was between 48 and 72 months, with a mean value of 60.72 ± 9.39 months (median = 60 months). The age range of the patients in the study group was 18 to 77 years old, with a mean value of 43.81 ± 11.5 years. The study group included 66 women (71.1%) and 26 men (28.9%). The mean age of the healed patients was 43.70 ± 11.67 years old (median = 44 years), and the mean age of the nonhealed patients was 47.00 ± 7.00 years old (median = 44 years). The age and gender (*p* > 0.05) and tooth type and tooth location (*p* > 0.05) had no statistically significant effects on the outcomes (Table 1).

Out of the 90 teeth, 87 (96.7%) were healed and 3 (3.3%) were nonhealed. Of the 3 nonhealed teeth, 2 of them had the PAI scores of 3 (lesion size between 2 and 5 mm) and 1 tooth had a PAI score of 5 (lesion size > 5 mm). The tooth with a PAI score of 5 was symptomatic, while the other two teeth were asymptomatic.

Based on the results of this study, there were no statistically significant differences between the preoperative, intraoperative, and postoperative prognostic factors and the outcomes (*p* > 0.05) (Table 3).

The preoperative PAI scoring and preoperative radiolucency were used as verification methods for each other. Both methods had a different approach to measure the same parameter: preoperative lesion size. The PAI scoring method used a scoring system to classify periapical lesions according to their sizes, while preoperative radiolucency used a measurement method in millimeters based on the lesion diameter. The agreement percentage between the two methods was 92.2% (*n* = 83). The kappa score of the agreement between the methods was 0.834 [95%confidence interval (CI) = 0.716‐0.953, *p* < 0.001]. Moreover, Gwet's agreement coefficient (AC) score was also calculated as a kappa score verification method, with a result of 0.898 (95%CI = 0.824‐0.973, *p* < 0.001).

The presence of postoperative signs and symptoms was significantly higher in the nonhealed cases (*p* = 0.033). Additionally, the presence of postoperative radiolucency was also significantly higher in the nonhealed cases (*p* = 0.002) (Table 3).

## 4. Discussion

Based on the results of the 4 to 6-year follow-up of the cases undergoing a single-visit root canal treatment with apical enlargement, the success rate was found 96.7%. A minimum 4-year evaluation was chosen according to European Society of Endodontology consensus report to make sure that the root canal treatment is either healed or nonhealed [28]. Although the primary purpose of this study was to evaluate the clinical outcomes of a single-visit root canal treatment with apical enlargement, the preoperative, intraoperative, and postoperative factors were also recorded in order to evaluate correlations between these factors and the outcomes. However, no significant correlations were found between any of the prognostic factors and the outcomes after the 4 to 6-year follow-up. These results may have been due to the low number of nonhealed cases in the study group.

Although an inception cohort is more preferable than a survival cohort, due to high number of dropouts and nonresponding subjects, it was not possible to include all subjects within the inception cohort and the lost was above that was required for high level of evidence [29]. The high number of lost subjects suggested the study group to be rather heterogeneous and transient which is admissible in a big city like İstanbul with many commuters, immigrants, and national and international visitors. However, the difference between the study group and the inception cohort was not significant which indicates that the study group was an acceptable representation of the inception cohort with no response bias.

The importance of apical patency and maintaining apical constriction for ideal root canal cleaning has been addressed frequently in the literature [4, 30–32]. Different approaches to gaining apical patency have been suggested by several authors [5, 10, 24, 31]. For example, some authors have suggested using a file that matches the size of the apical foramen in order to remove the debris [5, 24], while the others have advocated for using a file with a size smaller than that of the apical foramen in order to maintain apical patency (i.e., #10–15 files). The smaller size files were advocated to prevent the embolus effect of a similarly sized file and reduce further apical debris extrusion [10, 31, 33]. It has also been suggested that instead of using a mechanical approach, apical patency and debris removal should be achieved with abundant irrigation [34] and intracanal dressing [35], as even the penetration of a #15 file through the main foramen can cause apical transportation [36] or the buildup of cementum and dentinal chips at the apex [37]. However, in teeth with necrotic pulp accompanied with periapical lesions, bacteria is present beyond the apical constriction [38] and within the lesion itself [39]. During the irrigation procedure, extrusion of the solution beyond the apical constriction is avoided, where the infected cemental walls are present [24, 40]. Moreover, the irrigation solution efficacy decreases drastically due to the presence of apical debris [24]. It is unlikely that the microorganisms within the cemental root canal beyond apical constriction would be eliminated with only the chemical reactions of irrigation solutions. Therefore, mechanical cleaning should be encouraged over chemical cleaning, or both should occur simultaneously [24]. Unfortunately, achieving apical patency with a small-sized file or abundant irrigation that does not touch the infected divergent cemental walls does not necessarily mean the cemental wall has been cleaned [24]. Therefore, in necrotic tooth cases with periapical infections, apical enlargement has been suggested to disinfect this region [24]. Moreover, in single-visit treatments, due to the time constrictions, this method could be useful for instant and active apical cleaning when compared to chemical cleaning or the passive introduction of a smaller file through the apical foramen [24].

In an apically enlarged tooth, the root canal sealer may come in contact with periodontal tissues, which will delay the wound healing due to its chemical and toxic properties [41]. The highest root canal treatment success rate was achieved by finishing root canal treatment at the apical constriction, short of the radiographic terminus of the canal, although the localization of the apical constriction was still a matter of debate [10]. In two rat studies, large apical preparations were related with faster radiographic repair of the lesions [42, 43]. In another study, chronic apical lesion healing in dogs' teeth was reported to be more favorable in teeth with apical enlargement [5]. Large apical preparations were associated with better cleanliness of the root canal and less untouched places within the root canal system in extracted teeth with curved root canals [44]. These results were compatible with the results of the present study.

Yared and Dgaher [45] have reported that apical enlargement, conducted with either size 25 or size 40 files, 0.5 mm short of apex prior to intracanal Ca(OH)_2_ dressing between the appointments had significant difference on bacterial reduction. Souza et al. [46] reported that root canal instrumentation which was carried out by undergraduate students, either up to 3 or up to 4 following the initial file, 1 mm short of radiographic apex with intracanal Ca(OH)_2_ dressing showed no significant difference in regard to periapical healing after a 2-year follow-up. Saini et al. [23] reported no significant difference between root canal treatments which were prepared 2, 3, 4, 5, and 6 sizes larger than the first apical binding file after a 12-month follow-up. All root canal treatments were completed in two appointments with Ca(OH)_2_ intracanal dressing in between [23]. However, a meta-analysis in which Ca(OH)_2_ was used as intracanal dressing in all the included studies, it was reported that root canal treatments with larger apical sizes had increased healing in teeth with necrotic pulps and periapical lesions compared to smaller apical sizes [47]. One other meta-analysis reported that root canals obturated 0-1 mm from apex were statistically higher success rate compared to root canal obturated either >1 mm but <3 mm from apex or past the apex, further supporting the importance of apical shaping and working within 1 mm range of apical foramen [48].

The use of intracanal dressing which is not applicable in single-visit root canal treatments may not be compatible with the present study. Moreover, there are conflicting results between the studies due to the change in methods and operator skills [23, 45–48]. There is still no consensus on the treatment modality when it comes to single-visit versus multivisit treatments [17–20]. However, the high success rates, even in retreatment cases [19], and the presence of favorable conditions regarding periapical repair in single-visit cases, when compared to calcium hydroxide introduced multivisit cases [21], highlight single-visit root canal treatments as viable options for endodontic practice.

PAI scoring, which was first described by Ørstavik et al. [26], was used in the current study. This scoring system has been used in many longitudinal studies, and its significant prognostic value has been proven, particularly in repeated radiological evaluations, as described in previous studies [49, 50]. The absence of a radiolucent area on a radiograph does not equate to the absence of a lesion, and the precision of this method has been questioned with skepticism when compared to cone beam computed tomography (CBCT) [51]. On the other hand, the need for investigations of the radiographic interpretation of CBCT before its introduction in outcome studies in endodontics was recommended due to the significant variations in the periodontal ligament imaging in healthy teeth [52]. Therefore, periapical radiography and PAI scoring were chosen for the endodontic treatment follow-up in the present study. Although the prognostic strength of the full PAI scale has been demonstrated [49], in order to finalize the prognosis of the tooth, the PAI scores were dichotomized (the PAI scores of 1 and 2 were healed and the PAI scores of 3, 4, and 5 were nonhealed) as described in a previous study [19].

The preoperative evaluations of the periapical regions in the respective teeth were conducted using two different previously described methods [19]: the preoperative PAI scores and preoperative radiolucency (preoperative lesion size). Although they evaluate the same factor, they use different approaches. The preoperative PAI score evaluates the periapical region using a scoring method, while preoperative radiolucency evaluates the same factor by measuring the lesion size diameter on periapical radiographs. They were used to verify each other in order to further strengthen the reliability of both methods. The kappa score (0.834) and Gwet's AC score (0.898) showed very good agreement, indicating the interchangeability of the two methods in the preoperative evaluation of the periapical status. In one previous study, similar findings were reported regarding the agreement between these methods in terms of the preoperative periapical status evaluation [19].

Treading a thin line between destruction of apical constriction and reducing the complication risk due to the complexities of the apical anatomy, apical enlargement may prove to be a useful technique in disposal of the clinicians if applied accurately.

The need for more evidence-based research is evident in this area, and the promising long-term results of a single-visit root canal treatment with apical enlargement also provide a need for randomized controlled trials on this subject as well.

## 5. Conclusion

After a 60-month mean observation time, single-visit root canal treatments with apical enlargement on teeth with necrotic pulp tissue provided favorable outcomes, with a healing rate of 96.7%.

## Figures and Tables

**Table 1:** Distribution of the prognostic factors according to the inception cohort and study cohort, including the healed and nonhealed samples (*p* values).

	Inception cohort (*n* = 137)	Study cohort (*n* = 90)	Healed	Nonhealed	*p* value
	*n* (%)	*n* (%)	*n* (%)	*n* (%)	
Preoperative					
Gender					
Male	52 (38)	26 (28.9)	24 (92.3)	2 (7.7)	0.159^a^
Female	85 (62)	64 (71.1)	63 (98.4)	1 (1.6)	
Age at treatment					
<45 years old	67 (48.9)	47 (52.2)	45 (95.7)	2 (4.3)	0.625^a^
≥45 years old	70 (51.1)	43 (47.8)	42 (97.7)	1 (2.3)	
Preoperative pain					
Absent	76 (55.5)	53 (58.9)	52 (98.1)	1 (1.9)	0.611^a^
Present	61 (44.5)	37 (41.1)	35 (94.6)	2 (5.4)	
Tooth type					
Maxillary	86 (62.8)	51 (56.7)	49 (96.1)	2 (3.9)	0.358^a^
Mandibular	51 (37.2)	39 (43.3)	38 (97.4)	1 (2.6)	
Radiolucency					
Absent	3 (2.2)	2 (2.2)	2 (100)	0 (0)	0.681^b^
<2 mm	39 (28.5)	26 (28.9)	26 (100)	0 (0)	
2–5 mm	64 (46.7)	36 (40.0)	34 (94.4)	2 (5.6)	
>5 mm	31 (22.6)	26 (28.9)	25 (96.2)	1 (3.8)	
Preoperative PAI score					
1	1 (0.7)	1 (1.1)	1 (100)	0 (0)	0.310^b^
2	14 (10.2)	3 (3.3)	3 (100)	0 (0)	
3	88 (64.2)	57 (63.3)	55 (96.5)	2 (3.5)	
4	17 (12.4)	13 (14.4)	13 (100)	0 (0)	
5	17 (12.4)	16 (17.8)	15 (93.8)	1 (6.3)	
Periodontal defects					
Absent	131 (95.6)	85 (94.4)	82 (96.5)	3 (3.5)	0.757^a^
Present	6 (4.4)	5 (5.6)	5 (100)	0 (0)	
Intraoperative					
Root filling length					
Adequate	137 (100)	90 (100)	87 (96.7)	3 (3.3)	—
Short	0 (0)	0 (0)	0 (0)	0 (0)	
Long	0 (0)	0 (0)	0 (0)	0 (0)	
Root filling voids					
Absent	137 (100)	90 (100)	87 (96.7)	3 (3.3)	—
Present	0 (0)	0 (0)	0 (0)	0 (0)	
Complications					
No	134 (97.8)	87 (96.7)	84 (96.6)	3 (3.4)	0.684^a^
Yes	3 (2.2)	3 (3.3)	3 (100)	0 (0)	
Sealer extrusion					
No	76 (55.5)	49 (54.4)	49 (100)	0 (0)	0.879^a^
Yes	61 (45.5)	41 (45.6)	38 (92.7)	3 (7.3)	
Postoperative					
Density of root filling					
Dense and tapered	90 (100)	90 (100)	87 (96.7)	3 (3.3)	—
Voids present	0 (0)	0 (0)	0 (0)	0 (0)	
Poorly condensed	0 (0)	0 (0)	0 (0)	0 (0)	
Quality of coronal restoration					
Adequate	87 (96.7)	87 (96.7)	84 (96.6)	3 (3.4)	—
Marginal deficiency	3 (3.3)	3 (3.3)	3 (100)	0 (0)	
Postoperative signs/symptoms					
Absent	89 (98.9)	89 (98.9)	87 (97.8)	2 (2.2)	—
Present	1 (1.1)	1 (1.1)	0 (0)	1 (100)	
Radiolucency					
Absent	87 (96.7)	87 (96.7)	86 (98.9)	1 (1.1)	—
Present	3 (3.3)	3 (3.3)	1 (33.3)	2 (66.7)	
Postoperative PAI score					
1	68 (75.6)	68 (75.6)	68 (100)	0 (0)	—
2	19 (21.1)	19 (21.1)	19 (100)	0 (0)	
3	3 (3.3)	3 (3.3)	0 (0)	3 (100)	
4	0 (0)	0 (0)	0 (0)	0 (0)	
5	0 (0)	0 (0)	0 (0)	0 (0)	
Fracture					
Absent	90 (100)	90 (100)	87 (96.7)	3 (3.3)	—
Present	0 (0)	0 (0)	0 (0)	0 (0)	
Restoration at follow-up					
Definitive filling	43 (31.4)	30 (33.3)	28 (93.3)	2 (6.7)	0.759^a^
Crown	94 (68.6)	60 (66.7)	59 (98.3)	1 (1.7)	
Post					
Absent	93 (67.9)	61 (67.8)	58 (95.1)	3 (4.9)	0.987^a^
Present	44 (32.1)	29 (32.2)	29 (100)	0 (0)	

PAI: periapical index. ^a^Fisher's exact test. ^b^Fisher-Freeman-Halton exact test.

**Table 2:** Interexaminer (E.E. versus K.O.) and intraexaminer (1 versus 2) Cohen's kappa values according to the PAI scores that were recorded from the same radiographs with a 2-month interval.

PAI	Intraexaminer		Interexaminer	
	E.E.1-E.E.2	K.O.1-K.O.2	E.E.1-K. O.1	E.E.2-K.O.2
	*n* (%)	*n* (%)	*n* (%)	*n* (%)
1	23 (23.2)	22 (22.4)	22 (23.2)	22 (22.4)
2	24 (24.2)	26 (26.5)	24 (25.3)	24 (24.5)
3	19 (19.2)	18 (18.5)	18 (18.9)	18 (18.4)
4	20 (20.2)	20 (20.4)	19 (20)	22 (22.4)
5	12 (12.1)	12 (12.2)	12 (12.6)	12 (12.2)
Cohen's kappa	0.987	0.974	0.936	0.987
*p* value	<0.001^∗∗^	<0.001^∗∗^	<0.001^∗∗^	<0.001^∗∗^

^∗∗^ *p* < 0.001. PAI: periapical index.

**Table 3:** Distribution of the prognostic factors and their significance on the healed and nonhealed groups (post hoc values).

	*n*	Healed (*n* = 87)	Nonhealed (*n* = 3)	*p* value	Post hoc power
		*n* (%)	*n* (%)		
Preoperative					
Preoperative pain					
Absent	53	52 (98.1)	1 (1.9)	0.566^a^	0.148
Present	37	35 (94.6)	2 (5.4)		
Radiolucency					
<2 mm	26	26 (100)	0 (0)	0.778^b^	0.172
2–5 mm	36	34 (94.4)	2 (5.6)		
>5 mm	26	25 (96.2)	1 (3.8)		
Preoperative PAI score					
1	1	1 (100)	0 (0)	0.751^b^	0.134
2	3	3 (100)	0 (0)		
3	57	55 (96.5)	2 (3.5)		
4	13	13 (100)	0 (0)		
5	16	15 (93.8)	1 (6.3)		
Periodontal defects					
Absent	85	82 (96.5)	3 (3.5)	0.999^a^	0.071
Present	5	5 (100)	0 (0)		
Root filling density					
Dense and tapered	90	87 (96.7)	3 (3.3)	—	—
Voids present	0	0 (0)	0 (0)		
Poorly condensed	0	0 (0)	0 (0)		
Length of root filling					
Adequate	90	87 (96.7)	3 (3.3)	—	—
Short	0	0 (0)	0 (0)		
Long	0	0 (0)	0 (0)		
Intraoperative					
Sealer extrusion					
No	49	49 (100)	0 (0)	0.091^a^	0.486
Yes	41	38 (92.7)	3 (7.3)		
Postoperative					
Restoration at follow-up					
Definitive filling	30	28 (93.3)	2 (6.7)	0.257^a^	0.238
Crown	60	59 (98.3)	1 (1.7)		
Postoperative signs/symptoms					
Absent	89	87 (97.8)	2 (2.2)	0.033^∗^^,a^	0.999
Present	1	0 (0)	1 (100)		
Radiolucency					
Absent	87	86 (98.9)	1 (1.1)	0.002^∗∗^^,a^	0.999
Present	3	1 (33.3)	2 (66.7)		
Post					
Absent	61	58 (95.1)	3 (4.9)	0.548^a^	0.228
Present	29	29 (100)	0 (0)		

^a^Fisher's exact test. ^b^Fisher-Freeman-Halton exact test.^∗^*p* < 0.05. ^∗∗^*p* < 0.01. PAI: periapical index.

## Data Availability

The patients' data used to support the findings of this study are restricted by the Istanbul Medipol University Ethical Board in order to protect patient privacy. Data are available from Istanbul Medipol University, for researchers who meet the criteria for access to confidential data according to the Turkish Personal Data Protection Law no. 6698.
